# Enhancing Genome-Enabled Prediction by Bagging Genomic BLUP

**DOI:** 10.1371/journal.pone.0091693

**Published:** 2014-04-10

**Authors:** Daniel Gianola, Kent A. Weigel, Nicole Krämer, Alessandra Stella, Chris-Carolin Schön

**Affiliations:** 1 Department of Animal Sciences, University of Wisconsin-Madison, Madison, Wisconsin, United States of America; 2 Department of Dairy Science, University of Wisconsin-Madison, Madison, Wisconsin, United States of America; 3 Department of Biostatistics and Medical Informatics, University of Wisconsin-Madison, Madison, Wisconsin, United States of America; 4 Department of Plant Breeding, Technical University of Munich, Weihenstephan Center, Munich, Germany; 5 Bioinformatics and Statistics Unit, Parco Tecnologico Padano, Lodi, Italy; China Agricultrual University, China

## Abstract

We examined whether or not the predictive ability of genomic best linear unbiased prediction (GBLUP) could be improved via a resampling method used in machine learning: bootstrap aggregating sampling (“bagging”). In theory, bagging can be useful when the predictor has large variance or when the number of markers is much larger than sample size, preventing effective regularization. After presenting a brief review of GBLUP, bagging was adapted to the context of GBLUP, both at the level of the genetic signal and of marker effects. The performance of bagging was evaluated with four simulated case studies including known or unknown quantitative trait loci, and an application was made to real data on grain yield in wheat planted in four environments. A metric aimed to quantify candidate-specific cross-validation uncertainty was proposed and assessed; as expected, model derived theoretical reliabilities bore no relationship with cross-validation accuracy. It was found that bagging can ameliorate predictive performance of GBLUP and make it more robust against over-fitting. Seemingly, 25–50 bootstrap samples was enough to attain reasonable predictions as well as stable measures of individual predictive mean squared errors.

## Introduction

A method for whole-genome enabled prediction of quantitative traits known as GBLUP, standing for “genomic best linear unbiased prediction”, was seemingly suggested first by Van Raden [1]–[2]. In GBLUP, a pedigree-based relationship matrix among individuals [3] is replaced by a matrix valued measure of genomic similarities constructed using molecular markers, such as single nucleotide polymorphisms (SNPs) [4]. This “genomic relationship” matrix or variants thereof [5]–[6], referred to as 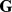 hereinafter, defines a covariance structure among individuals (even if genetically unrelated in the standard sense), stemming from “molecular similarity” in state at additive marker loci among members of a sample. Given 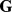 and values of some needed variance components, one can use the theory of best linear unbiased prediction (BLUP) to obtain point and interval (e.g., prediction error variances) estimates of the genetic values of a set of individuals as marked by the battery of SNPs. While 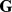 can be constructed in different manners we do not address this issue here. However, we note that it is possible to separate “genomic” from “residual” variance components statistically even in the absence of genetic relatedness. Hence, care must be exercised when relating the “genomic” to the “genetic” variance; this is discussed in [7].

Using theory developed by Henderson [8]–[10] it can be shown that GBLUP is equivalent to a linear regression model on additive genotypic codes of markers, with the allelic substitution effects at marker loci treated as drawn independently from a distribution possessing a constant variance over markers [11]–[13]. There is also an equivalence between pedigree-based BLUP or G-BLUP (or of models using both pedigree and marker relationships) and non-parametric regression [14]. For instance, if the 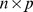 marker matrix 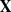 (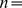
number of observations, 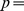
number of markers) is used to construct a kernel matrix 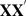, it can be established that GBLUP is a reproducing kernel Hilbert spaces regression procedure [15]–[16]. Also, BLUP and GBLUP can be represented as linear neural networks where inputs are entries of the pedigree-based or 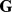 matrices, respectively [17]. Hence, GBLUP can be motivated from several different perspectives.

There are many competing procedures for genome-enabled prediction, such as the members of the growing Bayesian alphabet [14],[18]–[19], but most of these require a Bayesian Markov chain Monte Carlo (MCMC) implementation. On the other hand, GBLUP is simple, easy to understand, explain and compute, and there is software available for likelihood-based variance component estimation and for prediction of random effects. Also, GBLUP handles cross-sectional and longitudinal data flexibly and extends to multiple-trait settings in a direct manner. Further, GBLUP delivers a competitive predictive ability since members of the Bayesian alphabet typically differ by little in predictive performance and differences among methods are typically masked by cross-validation noise [19]–[23]. Last but not least, some members of this alphabet produce predictions that are sensitive to hyper-parameter specification [24]. Given these considerations, GBLUP or extensions thereof [25] are good candidates for routine whole-genome prediction in animal and plant breeding applications and possibly for prediction of complex traits in humans as well [7],[26].

The closeness between predictions and realized values depends mainly on three factors: prediction bias, variance of prediction error and amount of noise associated with future observations. The latter cannot be reduced by any prediction machine based on training data, so it is impossible to construct predictors attaining a perfect predictive correlation, even if the model holds. Theoretical and empirical results [1], [27]–[30] indicate that the proportion of (cross-validation) variance explained by a linear predictor increases up to a point with training sample size, then reaching a plateau. However, when a small number of individuals is available, any prediction machine is bound to produce predictions with a large variance. In this context, it seems worthwhile to explore avenues for enhancing accuracy (i.e., reduce bias) and reliability (i.e., decrease variance) of predictions when training size is small.

The question examined here is whether or not the predictive ability of GBLUP can be improved by recourse to resampling methods used in machine learning. These include bootstrap aggregating sampling (“bagging”) and iterated bagging or “debiasing” [31]–[34]. Bagging uses bootstrap sampling to reduce variance and can enhance reliability and reduce mean squared error [31]. The conditional bias of GBLUP [19] cannot be removed by bagging, but iterated bagging has the potential of reducing variance while removing bias simultaneously. This study investigates bagging of GBLUP, with consideration of debiasing deferred to a future investigation. The second section of this paper gives a review of GBLUP and of its inherent inaccuracy (bias). The third section describes bagging in the context of GBLUP at the level of the genetic signal and of marker effects. The fourth section examines the performance of bagging in four simulated case studies, and the fifth section presents an application to real data on grain yield in wheat planted in four environments. The paper concludes with a discussion and with a proposal of a metric aimed to quantify candidate-specific cross-validation uncertainty.

## Materials and Methods

### GBLUP

#### Idealized conditions

Assume that effects of nuisance factors (e.g., year to year variation) have been removed in a pre-processing stage (this can also be done in a single-stage, but we ignore this for simplicity). GBLUP can be motivated by positing the linear regression model on markers
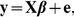
(1)where 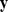 is an 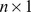 vector of observations or pre-processed data measured on a set of individuals or lines; 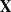 is an 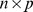 matrix of marker genotypes, with its typical element 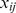 being the genotype code at locus 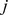 observed in individual 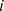, and with 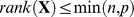; 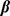 is a 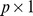 vector of unknown allelic substitution effects when marker genotypes are coded, e.g., as 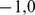 and 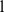 for 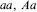 and 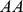 at locus 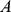, say, or when these coded values are deviated from the corresponding column means or standardized. Above, 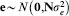 is a vector of residuals where 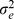 is the residual variance and 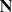 is an 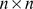 diagonal matrix with typical element 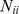; if 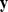 consists of single measurements on individuals, 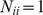 for all 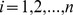, and if 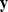 is a vector of means, 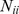 would be the number of observations entering into the mean. In BLUP, a distribution is assigned to 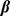 and the simplest one is 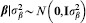, where 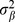 is the variance of marker allele substitution effects. Using this assumption together with model (1) gives as marginal distribution of the data (after assuming that 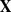 and 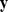 have been centered) 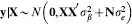. In BLUP 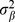 and 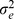 are treated as known but these parameters are typically estimated from data at hand [35]. With markers, most often 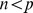 so it is convenient to form the best linear unbiased predictor of 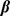 as 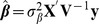, where 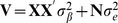. If model (1) holds, it can be shown that 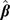 is unbiased in the sense that 
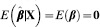
. Its covariance matrix (given 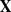) is
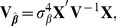
(2)and its prediction error covariance matrix (also covariance matrix of the conditional distribution of 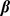 given 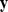 and 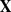 under normality assumptions) is
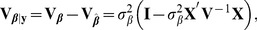
(3)where 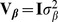. The diagonal elements of 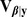 (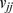) lead to a measure of reliability of prediction of marker effect 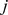: 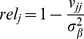. A matrix of reliabilities and co-reliabilities is
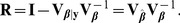
(4)If one wishes to predict a future vector 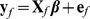, with future residuals independent of past ones and provided that future and past residuals stem from the same stochastic process, under normality assumptions the predictive distribution [19] is
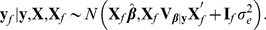
(5)Further, if 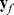 is the predictand and 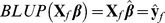 is the predictor, BLUP theory yields
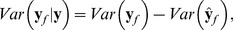
(6)so that 
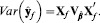
. If the marker effects could be estimated such that 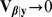, then 
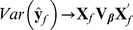
 and 
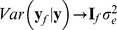
. Hence, the distribution in (5) would still have variance, indicating that it is not possible to attain a predictive correlation equal to 1 even if a training set has an infinite size (more formally, if the training process conveys an infinite amount of information about markers); [7] gives a discussion of related matters.

### Blup is conditionally inaccurate

While BLUP theory is well established, quantitative geneticists tend to interpret the unbiasedness property of BLUP as if it pertained to the true unknown 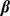, when in fact it applies to the average of the distribution of 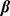, that is, 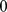. If 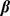 in (1) were viewed as a model on unknown effects of known quantitative trait loci (QTL), it is obvious that one should think in terms of a fixed effects model, as per the standard finite number of loci model of quantitative genetics [36]. Accordingly, if effects of markers are sought because these “flag” some genomic region of interest, the random sampling assumption made in BLUP is not relevant, although it might lead to a more stable estimator. In the fixed effects case both 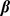 and the marked genetic signal 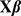 are estimated with bias by BLUP even if the model holds [19].

Markers are not QTL and the latter are “causes” of generating a signal to phenotype. Suppose that the “true model” is linear on effects 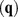 of QTL relating to phenotypes via incidence matrix 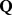, that is
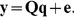
(7)If the QTL effects 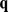 are viewed as fixed entities (arguably geneticists have this in mind in their quest of finding genes), 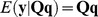. In this situation BLUP produces the following average outcomes
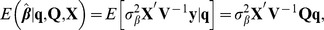
and
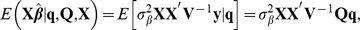
so the bias of the estimated signal is 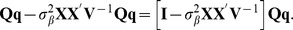


On the other hand, if 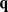 is assigned a distribution, say, 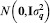 and the 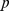 markers are treated as random as well, e.g., 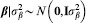, under normality assumptions one has
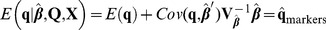
(8)where
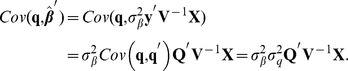
Using this in (8)
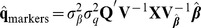
where 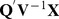 conveys unknown linkage disequilibrium relationships between QTL and marker genotypes; similarly, the best statement about the signal is 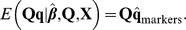 Unfortunately, neither 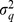 nor 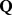 are known, so statements made about QTL from markers are based on untestable assumptions, including the view that the QTL effects and the genotypes are linearly related, as in (7).

### Predictive correlation when markers are the QTL

Imagine a best case scenario where the markers are the QTL, and consider predicting 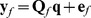. Here, 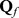 is the incidence matrix relating QTL effects to yet to be realized phenotypes 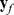, and 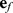 is a future vector of residuals. BLUP theory, using 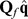 (here, 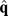 is the 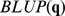 under the true model) as predictor, gives the following squared correlation between the 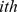 elements of 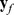 and 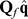 (below 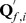 is the 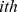 row of 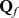)
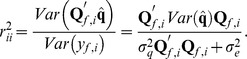
Let now 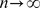 in which case BLUP theory yields 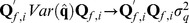, so that
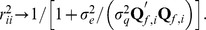
This shows that it is impossible to attain a perfect predictive correlation even when the markers are the QTL. Further, 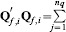 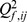, where 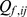 is the genotype at QTL locus 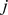 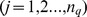 of individual 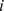 in the testing set. If QTL genotypes are centered and assumed to be in Hardy-Weinberg equilibrium 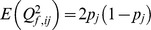, so approximately
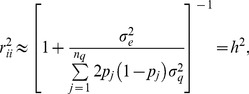
(9)where 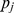 is the frequency of a reference allele, 
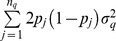
 is additive genetic variance and
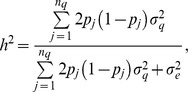
is trait heritability. Hence, the predictive correlation has an upper bound at 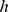.

If instead of predicting individual phenotypes the problem is that of predicting an average, the upper bound for the predictive correlation is higher. The corresponding formula is easy to arrive at and it just requires replacing 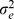 in (9) by 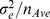, the number of observations intervening in the average. Then
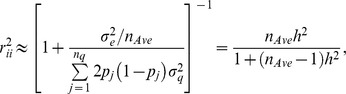
which is heritability as used in plant breeding, or “heritability of a mean” [36]. The predictive correlation never reaches 1 but can get close to 1 if 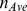 is very large. Values of squared predictive correlations in the range of 0.5–0.75 have been attained in dairy cattle progeny tests where predictands are processed averages of production records of cows sired by a bull [2].

### Bagging GBLUP (BGBLUP) and marker effects

#### Bagging GBLUP

“Bagging” exploits the idea that predictors can be rendered more stable by repeated bootstrapping and averaging over bootstrap samples, thus reducing variance [31]. Bagging has been found to have advantages in cases where predictors are unstable, i.e., when small perturbations of the training set produce marked changes in model training [31], [34], [37]; for example, with ordinary least-squares under severe multi-colinearity. An important application of bagging is in prediction using random forests [38].

Prediction methods that use regularization, such as those applied in genome-enabled selection, are often stable because penalties on model complexity reduce the effective number of parameters drastically, thus lowering variance. However, this is attained at the expense of bias with respect to marker effects and to the unknown function to be predicted (marked genetic value). A priori it would seem that bagging would not bring advantages in the context of a regularized method such as GBLUP. However, this issue has not been examined so far and there may be cases, e.g., in “small” populations, where random variation in training sets of small sizes has a marked impact on predictive ability.

To motivate bagging, we recall that GBLUP is a regression of phenotypes on genomic relationships between individuals. Let 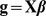 be a vector of “genomic values”, 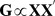 and assume that 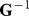 exists; then (1) can be written in equivalent form as
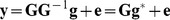
(10)where 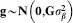 and 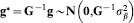. In scalar form, the datum for individual 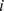 is expressible under this parameterization as the regression
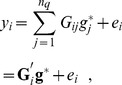
(11)where 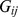 is an element of the 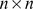 matrix 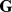 and 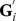 is its 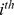 row. From basic principles,
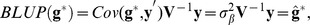
and since 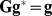, use of invariance gives
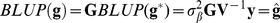
(12)Note that 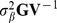 is an 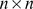 matrix of “heritabilities” and “co-heritabilities”; the diagonal elements of 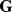 may be distinct from each other, so each individual may have a different heritability ascribed to it, as noted by de los Campos et al. (2013).

The intuitive idea behind bagging was outlined in [1]. Suppose there is a large number of training samples from the same population; by averaging over the predictions made from these samples we would end up with a reduction of variance but with the bias properties remaining the same as those from the predictor derived from a single training set. This large supply of training sets can be emulated by bootstrap sampling: by averaging over samples, one gets “closer” (in the mean squared error sense) to the true value, on average. Technical details of why this works are given in Appendix S1.

Let the predictor formed from a single training set of size 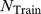 be 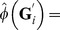 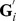 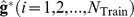. Its variance can be lowered by taking 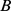 bootstrap copies of size 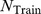 (i.e., sampling with replacement from the training set) and then averaging over copies. A given 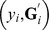 may not appear at all or may be repeated several times over the 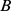 bootstrap samples. The bagging algorithm is:

- For each copy 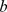 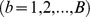 run GBLUP using estimates of variance components obtained from the entire data set (to simplify computations), find the regressions 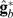 and form a bootstrap draw for 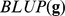 as
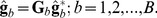
(13)
- After running the 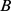 GBLUP implementations take the following averages
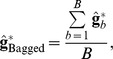
and
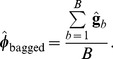
(14)
- Predict vector 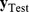 in the testing set as 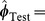 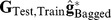 where 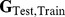 is a matrix of genomic relationships between individuals in testing and training sets. If 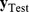 pertains to records on the same individuals the predictor is 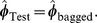

To see how improvement arises consider the following argument. We seek to learn signal 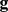 from the GBLUP predictor 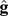. Under the setting of BLUP (where 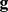 and 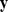 both vary at random over conceptual repeated sampling), the best predictor of 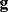 in the mean squared error sense is 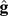 because this is the conditional expectation under normality [39]–[40]. However, if 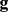 is the signal of a fixed target set of candidates, 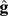 is biased as shown earlier. Thus, 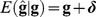, where 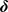 is a vector of biases, and the mean squared error matrix of 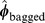 is
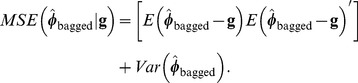
Now, if the 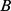 bootstrap copies of GBLUP are drawn from the same distribution, 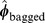 has the same expectation as 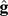 and, therefore, the same bias: 
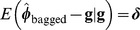
. Further, if the 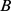 copies are viewed as drawn independently, the variance of 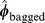 should be 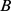 times smaller than that of any 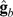. Thus
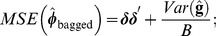
a formula for taking the correlation between samples into account is not available but the reduction in 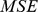 would be obviously smaller than in the idealized situation where samples are independent. Hence, 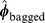 has the same bias of GBLUP but at best is 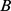 times less variable. If a prediction machine has little variance, as it is the case for shrinkage methods with a large amount of regularization, predictive performance might be degraded [31], but whether this occurs in a particular problem or not can be assessed empirically only.

### Bagging marker effects

Alternatively, one can work directly with the linear model (1), but this is more involved computationally because the problem becomes a 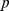-dimensional one (in GBLUP there are 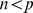 unknowns). Assuming centered data, the ridge regression estimator (BLUP of marker effects) is
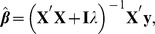
where some 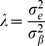 (in the BLUP framework) is available, estimated from data at hand or chosen over a cross-validation grid. Given the data matrix 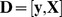, of order 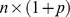, draw 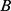 bootstrap copies by randomly sampling 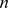 rows from 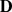 with replacement, such that a particular bootstrap sample is 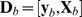. The bagged ridge regression estimator is
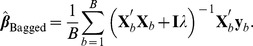
Then, given an out of sample case with marker matrix 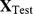, the yet-to be realized phenotypes are predicted as 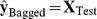 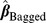.

Using the relationship 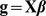, one can obtain “indirect” samples of the bootstrap distribution of 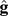 as 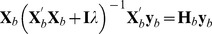 (where 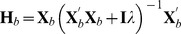 is the “hat” matrix for sample 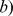 and form the “indirect” bagged GBLUP as
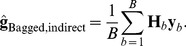
(15)


## Results

### Simulated case studies

#### Case Study 1: model training with 200 known QTL and 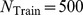


To evaluate the impact of bagging on model training, we simulated 200 QTL with known binary genotypes (as in inbred lines, where genotypes at a given locus are either 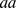 or 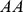). Genotypes were sampled with a frequency of 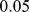 at all loci and their effects were drawn independently from a 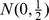 distribution; sample size was 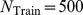 so all QTL effects were likelihood identified (estimable) in the regression model described below. Any resulting disequilibrium was due to finite sample size only. Phenotypes were formed by summing the product of QTL genotype codes (0,1) times their corresponding effects over the 200 QTL and then adding a random residual drawn from 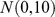; the “effective” heritability (variance among simulated realized genetic values as a fraction of the total variance) attained in the simulation was 0.34. The “true” model was employed in the training process using the “true” variance ratio 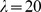 and the effect of the number of bootstrap samples 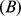, each of size 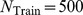, on the bagged predictor was examined by taking 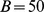, 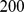 and 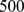. While 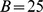 or 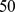 is often adequate [31], a larger number of bootstrap samples is not harmful.

Regressions of the 200 elements of 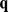 on either their ordinary least-squares estimates (OLS), BLUP-ridge regression 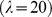 and on a bagged mean obtained by averaging over bootstrap sample estimates of the BLUPs of 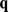 were calculated. This was also done for the regression of the 500 simulated genetic signals 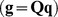 on either GBLUP and on the average of the GBLUP bootstrap samples 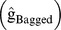. Table 1 presents results. As expected from BLUP theory [10] the regressions of either 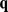 or 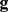 on their corresponding BLUPs were near their expected value: 1. By construction, 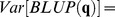 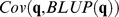, so the expected regression is necessarily 1. For QTL effects, OLS, even though being an unbiased estimator of 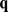, produced a regression of about 0.42. The bagging procedure produced regressions that exceeded 1, both at the level of the QTL effects and of the genetic signal 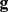. From the point of view of goodness of fit to the data, ridge regression and bagging produced models that accounted for more variation of the training data than OLS: for OLS 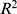 was about 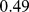 whereas it ranged between 0.51 and 0.53 for bagging and ridge regression. Increasing the number of bootstrap copies in the bag increased 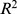 mildly with no sizable gain resulting from increasing 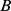 from 200 to 500. The regressions of 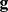 on 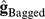
were larger than 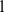 but the bagged means accounted for the same proportion of variation in true 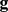 values as 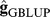 did, with 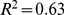 for the latter and for bagged GBLUP with 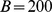 or 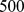.

**Table 1:** Regression coefficients of true QTL effects 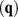 on their ordinary least-squares 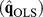, ridge regression BLUP 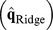 and bagged ridge regression BLUP estimates 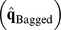, and regressions of true genetic signal 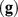 on genomic BLUP 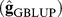 and bagged genomic BLUP 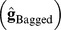 at varying number of bootstrap samples 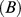.

	No. Bootstrap samples 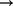	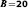	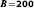	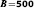
Regressions of 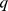 on 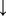				
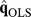	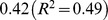	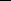	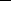	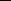
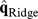	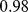 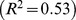	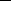	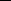	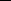
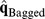	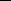	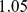 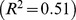	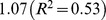	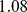 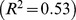
Regressions of 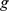 on 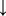				
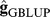	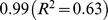	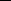	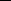	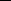
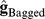	-	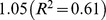	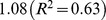	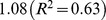

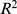
 is the coefficient of determination of the regression fitted. The simulation involved a training set of 500 individuals with 200 true additive QTL fitted in the model.

**Figure 1:**
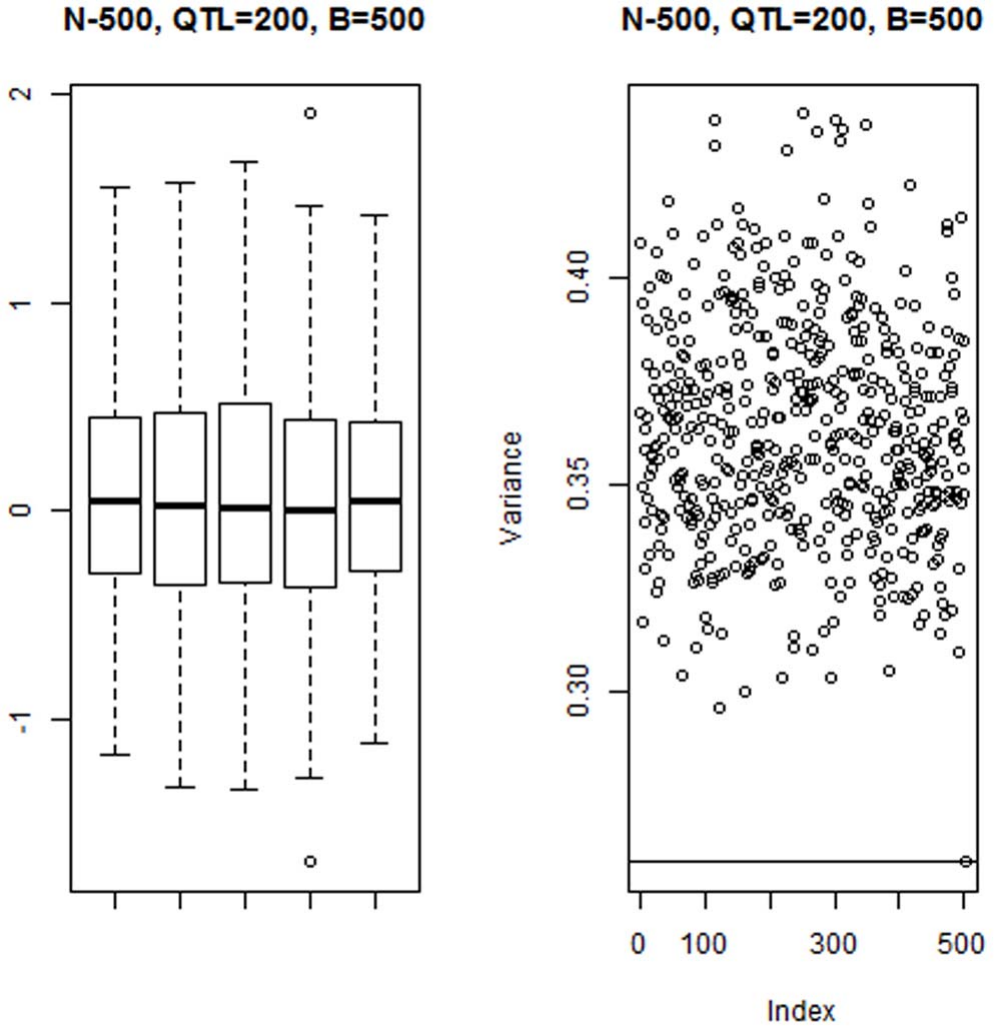
Simulation with 200 known QTL, 500 individuals in the training sample and 500 bootstrap copies for bagging. Left panel: distribution of 200 effects within each of 4 bootstrap samples (1–4), and within the average (bag) of 500 samples (5). Right panel: distribution of variance among 200 effects within each of 500 bootstrap samples and within their average (item 501, flagged with arrow).

**Figure 2:**
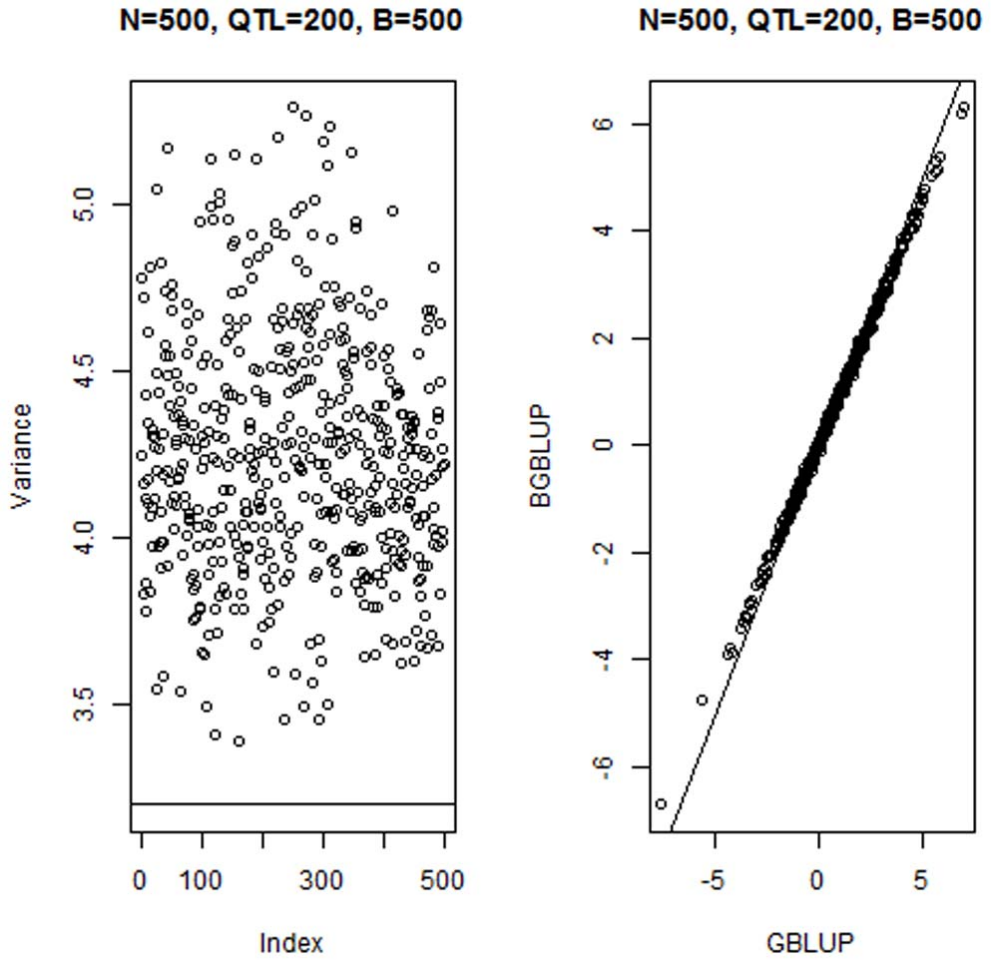
Simulation with 200 known QTL, 500 individuals in the training sample and 500 bootstrap copies for bagging. Left panel: variance (VAR) among 500 GBLUPs in each of 500 bootstrap samples. The horizontal line gives the variance among bootstrap (bagged) means for the 500 individuals. Right panel: scatter plot of bagged GBLUP (BGBLUP) versus exact GBLUP for 500 individuals.

Figure 1 (left panel) gives the distribution of estimates of the 200 QTL effects within each of 4 bootstrap samples for the run with 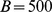 as well as within the vector of bagged means. As expected bagging produced less variability among individual effect estimates, as “extreme” values are tempered by the averaging procedure. The impact of bagging is seen in the right panel of Figure 1: the variance among bagged means of individual effects was much less than the variance within any of the 500 bootstrap copies. Figure 2 (left panel) shows the variance reduction when bagging was applied to the GBLUPs of individuals: the horizontal line at the bottom is the variance among the bagged GBLUPs of the 500 individuals in the training sample. The right panel of Figure 2 shows that bagged GBLUP (BGBLUP) produces an understatement of genetic values relative to what is predicted by GBLUP for individuals ranked as “high” by the latter, but an overstatement otherwise; however, the two procedures give aligned predictions of genetic values. Bagging regresses extreme GBLUP estimates towards their average, which might attenuate influence from idiosyncratic samples on which GBLUP is trained. Hence, bagging enhances the shrinkage towards 0 inherent to GBLUP.

#### Case Study 2: model training with 1000 known QTL and 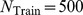


As in case 1, sample size was 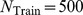 but 1000 QTL with known binary genotypes and unknown effects were simulated. Here 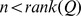 so least-squares cannot be used due to lack of estimability. The setting was as in case 1, but “effective heritability” was 0.68, that is, twice as when 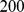 QTL were simulated. The “true” model was employed in the training process using the “true” variance ratio 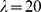, and the number of bootstrap samples for bagging was 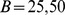 or 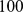.

**Figure 3:**
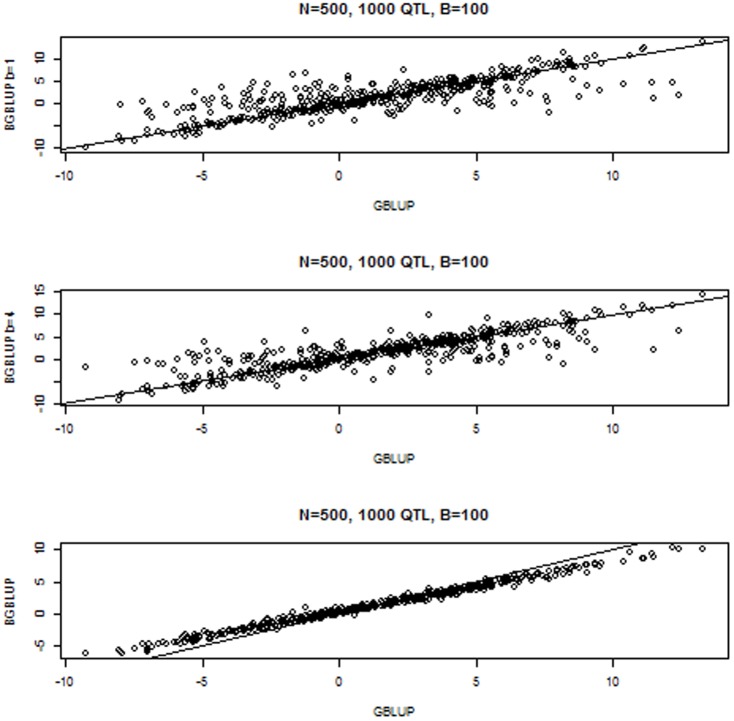
Simulation with 1000 known QTL, 500 individuals in the training sample and 100 bootstrap copies for bagging. Left panel: relationship between GBLUP with the entire sample and GBLUPs in 4 bootstrap samples of size 500. Right panel: relationship between bagged GBLUP (BGBLUP) and GBLUP of the 500 individuals.

Results are presented in Table 2. The regressions of 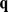 on 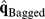 were much larger than 1 and the slope seemingly stabilized at about 1.32 with a bag consisting of 50 bootstrap copies. As expected, the regression of 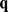 on 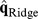 was near 1. However, the proportion of variation in true 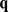 accounted for by the variation in bagged or ridge regression estimates was smaller than in case 1, with 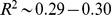 for bagging and ridge regression versus 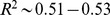 in case 1. On the other hand, bagged GBLUP accounted for about 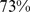 of the variation of the true genetic values 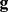, providing a “fit to signal” similar to that of GBLUP. The regression of 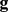 on 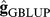 was also near its expected value of 1, whereas true differences in genetic values among individuals were exaggerated by a factor of about 1.25–1.27 by bagging. The left panel in Figure 3 shows the alignment between 4 randomly chosen bootstrap samples and the 500 GBLUP estimates obtained in 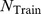. The right panel illustrates clearly that bagging 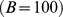 “pulls down” individuals with large GBLUP values and “pulls up” those placed at the left tail of the distribution of GBLUPs.

**Table 2:** Regression coefficients of true QTL effects 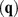 on their ridge regression BLUP 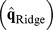 and bagged ridge regression BLUP estimates 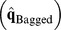, and regressions of true genetic signal (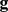) on genomic BLUP 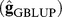 and bagged genomic BLUP 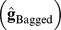 at varying number of bootstrap samples 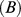.

	No. Bootstrap samples 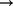	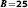	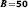	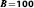
Regressions of 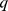 on 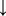				
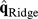	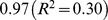	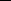	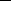	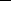
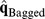	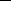	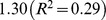	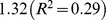	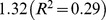
Regressions of 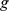 on 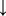				
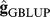	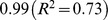	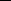	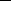	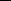
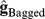	-	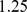 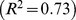	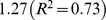	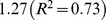

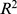
 is coefficient of determination of the regression fitted.

The simulation involved a training set of 500 individuals with 1000 true additive QTL fitted in the model.

#### Case Study 3: model training with 20 unknown QTLs, 200 markers and 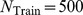


The setting was as in case 1 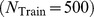 but here the genetic signal was generated from 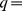
20 QTL with unknown binary genotypes that were in linkage disequilibrium (LD) with 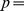
200 binary markers as specified below. The true model was
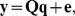
where 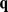 is a 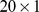 vector of allelic substitution effects. QTL genotypes were equally frequent at all loci and their effects were drawn independently from a 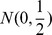 distribution. Phenotypes were formed by summing the product of QTL genotype codes (0,1) times their corresponding effects over the 20 QTL, and adding a random residual drawn from 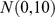. The method employed for training was ridge regression (BLUP) on 200 markers using the “true” variance ratio 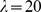, and the number of bootstrap samples for bagging was 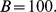 The simulation generated an effective heritability of about 0.19.

LD was simulated statistically by introducing correlations among columns of the 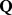 (QTL genotypes) and 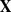 (marker genotypes) matrices, respectively. This was done by drawing 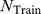 independent 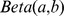 random variables corresponding to the rows of these matrices and then sampling a Bernoulli random variable (i.e., 0 for 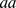 and 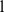 to 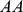, say) with probability of success given by the draw from the 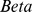 distribution. Thus, columns of matrices 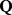 or 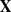 (with entries 0 or 1) had a beta-binomial distribution (e.g., Casella and George, 1992) and an expected correlation equal to 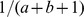; employing 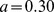 and 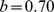 a correlation equal to 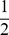 would be expected. Using this approach the “first” 10 QTL were in LD among themselves as well as in LD with the “first” 100 markers; these markers were in mutual LD themselves with a correlation of 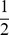 also. On the other hand, QTL 11–20 were in mutual linkage equilibrium as well as with all other markers. To illustrate, in the sample simulated realized genotypes at QTL 1 and 2 had a correlation of 0.48, whereas QTL 1 and 15 had a correlation of −0.02. Likewise, the correlation between markers 1 and 2 was 0.50, that between markers 1 and 200 was −0.04 and QTL 2 was correlated with marker 105 at 0.03. QTL genotypes at each locus were multiple-regressed on the 200 marker genotypes: for QTL 1–10 (in LD with markers) the 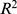 of the regression of QTL genotypes on the 200 marker genotypes was about 0.70 or larger. For the 10 QTL in LE with markers 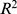 fluctuated around 0.40. This last result illustrates that even a null association accounts for some variation, merely because the likelihood increases monotonically with model complexity (in this case there are 200 partial regressions of each QTL genotype on markers).

The regression of phenotypes on QTL genotypes or on markers had an 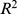 at 0.24 and 0.43, respectively, with the latter being larger simply because more parameters are fitted in the model. On the other hand, the squared correlation between true signal and fitted values was 0.87 for the QTL model versus 0.15 for the marker-based model: even though markers captured more variation (because of higher model complexity) than a regression on true genotypes, their ability of capturing signal was much less.

**Figure 4:**
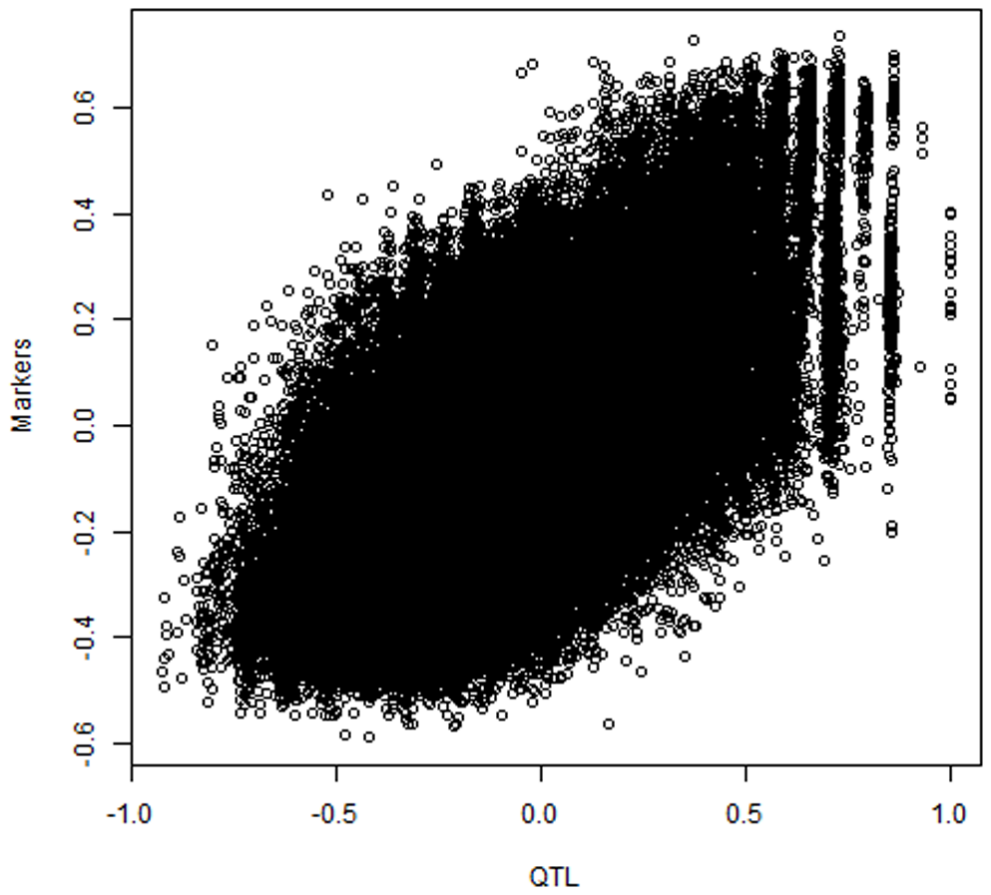
Simulation with 20 unknown QTL, 200 markers and 500 individuals: “genomic correlations” among 500 individuals at QTL or marker loci.

We measured similarity among individuals by constructing “genomic correlation matrices”. This was done by centering both 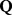 and 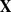, calculating 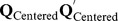 and 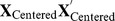, and converting these into correlation matrices 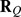 and 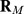, respectively. A plot of the off-diagonal elements of 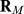 versus those of 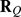 is shown in Figure 4 for the corresponding pairs of individuals. Although there is an association between genomic correlations at the QTL and marker levels, the latter ones were smaller in absolute values. This association was not perfect: at a given level of correlation at the QTL level, there was much variation in relationships when measured by markers. Implications of this on accuracy of genome-enabled prediction are discussed in [7].

GBLUP and BGBLUP were calculated at each of 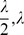 and 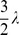 (with 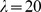) as variance ratio, to investigate the impact of regularization on the ability of capturing “true” signal, 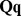. The regression of signal on GBLUP was 0.48, 0.54 and 0.60 for the three values of the regularization parameter, respectively, whereas that for BGBLUP was 0.50, 0.59 and 0.65, respectively. This illustrates that the regression of “true values” on GBLUP predictions is not 1 when the model is incorrect, as it is the case when markers are not QTL, as simulated here. The corresponding 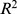 values were 0.25, 0.27 and 0.28 for GBLUP, and 0.24, 0.28 and 0.30 for BGBLUP. While 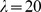 is the “correct” regularization to be exerted at the QTL level, this is not so for the model based on markers, where a stronger degree of regularization is needed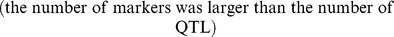. An approximation is that the “correct” variance ratio for the marker based model should be 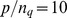 times larger than 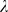, where 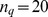 is the number of QTL. We found (results not shown) that the regressions of signal on GBLUP and BGBLUP increased as larger values of the regularization parameter were applied to the marker based model, with the “optimum” being near 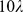, as expected.

#### Case Study 4: predictive cross-validation with 100 unknown QTLs and 500 markers

The simulation posed 100 unknown QTL whose additive effects were drawn from a 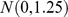 distribution and 500 individuals genotyped for 500 markers. The LD structure was similar to that of case 3: the first 50 QTL were in mutual linkage disequilibrium as well as in LD with the first 250 markers. QTL 51–100 were in LE among themselves as well with all markers; the first 250 markers were in mutual LD and markers 251–500 were in LE. Residuals were drawn from 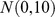 and the “effective” heritability attained was about 0.74.

The 500 individuals were distributed at random into two non-overlapping training and testing sets with 250 members in each. This was done 100 times at random, producing 100 training-testing pairs enabling estimation of the cross-validation distribution. GBLUP and BGBLUP were fitted to the training data 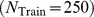 for each of 13 values of the regularization parameter 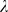 in the sequence
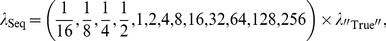
(16)where 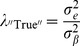 with 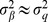 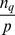. In each training instance, BGBLUP was implemented with 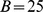 bootstrap samples of size 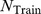 drawn from the training set of size 250. Three metrics were used to evaluate the two prediction methods: goodness of fit in the training set (correlation between fitted and observed values), predictive correlation (predicted phenotypes in the testing set and realized values) and predictive mean-squared error, that is, average squared difference between predicted and realized values over the 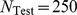 cases in the testing set.

**Figure 5:**
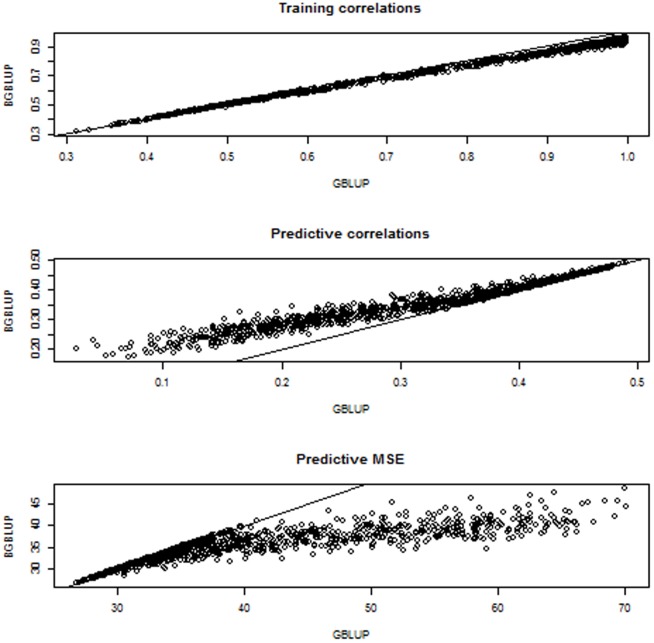
Simulation with 100 unknown QTL, 500 markers and 250 individuals in each training and testing set: training correlations, predictive correlations and predictive mean-squared error (MSE) for 1300 comparisons between bagged GBLUP (BGBLUP, 25 bootstrap samples) and GBLUP.

The preceding involved 13 BGBLUP and GBLUP implementations in each of the 100 random cross-validations, for a total of 1300 comparisons. Overall (Figure 5), BGBLUP attained a better predictive performance than GBLUP because predictive correlations were typically larger (in some cases more than twice as large as GBLUP) and mean squared errors of prediction were lower as well. Thus, BGBLUP was more reliable (larger correlation) and more accurate (smaller mean squared error) than GBLUP. The superiority of BGBLUP over GBLUP became smaller when regularization was stronger over the grid defined by 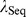. However, it was not until 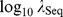 became 5 or more times larger than (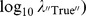 that GBLUP “caught up” with bagging but was never better. Actually, it was not until the 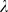 value used for model training was 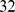 times larger than 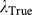 that the two predictors delivered the same performance, suggesting a “robustness” property of BGBLUP that GBLUP seems to lack. Model complexity is reduced as 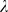 increases (the effective number of parameters decreases) so the variance of GBLUP decreases as well, in which case bagging offers little help. Contrary to what was stated by [7] we did not find evidence that bagging damaged predictive performance under any of the regularization regimes entertained. Results for selected settings are discussed in the following paragraph.

**Figure 6:**
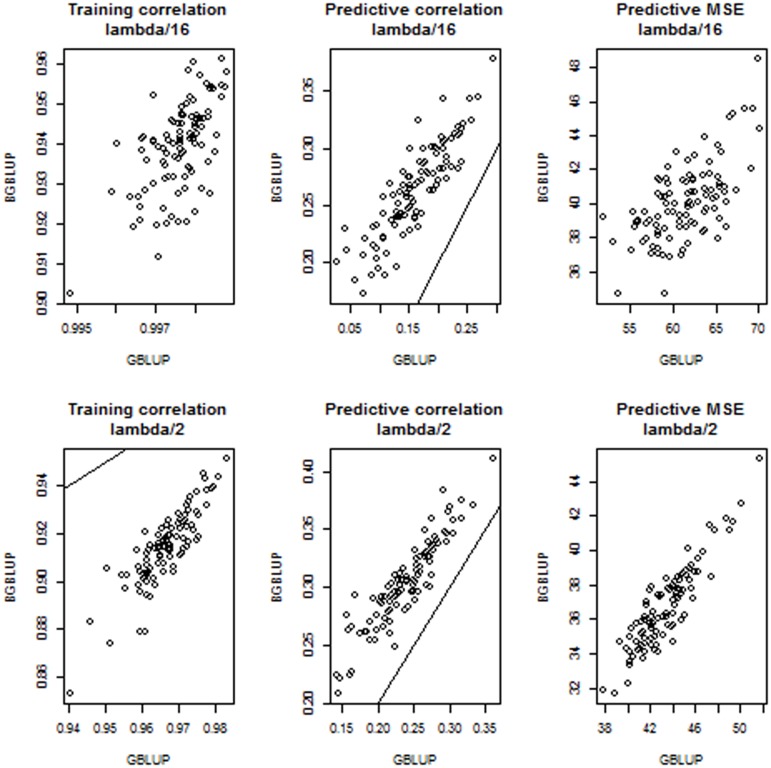
Simulation with 100 unknown QTL, 500 markers and 250 individuals in each training and testing set: training correlations, predictive correlations and predictive mean-squared error (MSE) for 100 comparisons between bagged GBLUP (BGBLUP, 25 bootstrap samples) and GBLUP at two levels of “under-regularization”.

Figure 6 shows training and predictive correlations and mean squared errors for BGBLUP (y-axis) and GBLUP (x-axis) at 2 levels of “under-regularization”: 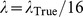 and 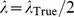. Here, where shrinkage is less than it should be, given the model, BGBLUP reduced overfitting (smaller training set correlations), increased predictive correlations and reduced mean squared errors, relative to BLUP. Differences were marked: in the upper (lower) middle panels of Figure 6 it is seen that the largest predictive correlation attained by GBLUP was smaller than 0.30 (0.39) and that bagging increased the corresponding correlation to about 0.38 (0.41). The effect of bagging on reducing predictive mean squared was also clear.

**Figure 7:**
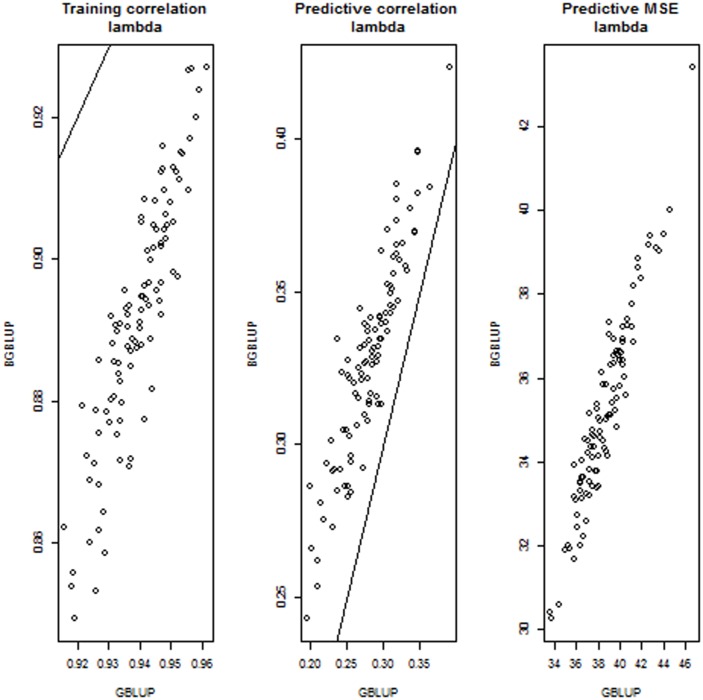
Simulation with 100 unknown QTL, 500 markers and 250 individuals in each training and testing set: training correlations, predictive correlations and predictive mean-squared error (MSE) for 100 comparisons between bagged GBLUP (BGBLUP, 25 bootstrap samples) and GBLUP at the “correct” level of regularization.

**Figure 8:**
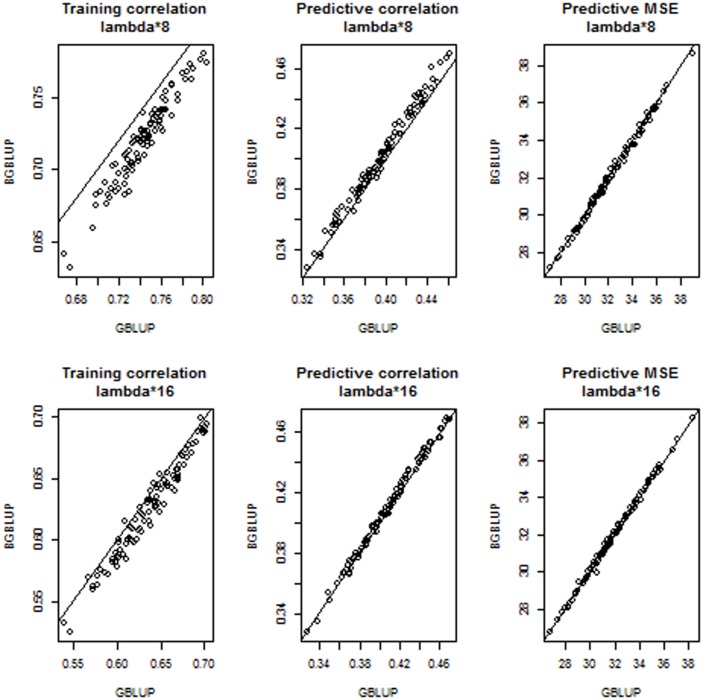
Simulation with 100 unknown QTL, 500 markers and 250 individuals in each training and testing set: training correlations, predictive correlations and predictive mean-squared error (MSE) for 100 comparisons between bagged GBLUP (BGBLUP, 25 bootstrap samples) and GBLUP at two levels of “over-regularization”.

Figure 7 presents results when the “true” value of 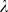 (400) was used for training. GBLUP was again close to overfitting and BGBLUP reduced training correlations, thus tempering the problem. Predictively, BGBLUP was better than GBLUP in all 100 comparisons, both in the correlation and MSE senses. Over the 100 cross-validation runs, the predictive correlation ranged between 0.195 and 0.391 (median 0.282) for GBLUP and between 0.243 and 0.423 (median 0.330) for BGBLUP. MSE of prediction ranged between 33.64 and 46.66 (median 38.47) for GBLUP and between 30.24 and 43.41 (median 35.01) for BGBLUP. Comparing the medians of the distributions, BGBLUP enhanced the predictive correlation by 17% and MSE was 91% of that of GBLUP. Figure 8 depicts what was found when “excessive” shrinkage was applied in training: the regularization parameter values were 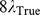 (upper panel) and 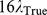 (lower panel). BGBLUP was only marginally better than GBLUP. Strong shrinkage rendered the training model exceedingly simple so both methods delivered similar same predictive ability. Differences between methods vanished when 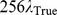 was used as shrinkage parameter.

We repeated the experiment with the setting of case study 4 but increasing training and testing sample sizes to 2500 each. Here, training sample size was 5 times larger than the number of markers: no differences between BGBLUP and GBLUP were found at any level of regularization. The reason is that 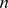 now exceeds 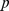, making GBLUP fairly stable, in which case the variance reduction property of BGBLUP does not help much.

In summary, in our simulations BGBLUP was typically better than GBLUP at most points of the regularization grid considered. Its performance was better than that of GBLUP at values close to “optimal” regularization, and differences were large when shrinkage was small, because bootstrap sampling with averaging reduced variance. If 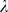 is small, GBLUP tends to overfit and to be variable but BGBLUP alleviates these problems. In addition to helping with overfitting, BGBLUP was robust with respect to departures from optimal regularization, e.g., to errors in the variance ratio. The experiment with a much larger training sample size than the number of markers indicated that the performance of bagging depends on 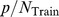: we conjecture that as this ratio increases (as it will surely be the case with DNA sequence data) use of BGBLUP may enhance predictive ability in some real data situations, simply because overfitting and colinearity will be exacerbated by introducing a massive number of variates in the model.

### Analysis of wheat data

The data set is at http://cran.r-project.org/web/packages/BLR/index.html, in the BLR package in 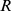, and has been used by, e.g., [17],[42]–[43]. The data represents 599 wheat inbred lines each genotyped with 1279 DArT (Diversity Array Technology) markers, and planted in 4 distinct environments. DArT markers may take on one of two values, denoting presence or absence of an allele. Records came from several international trials conducted at the International Maize and Wheat Improvement Center (CIMMYT), Mexico. The trait considered was average grain yield for each line in each of the four environments. This response variable is an average over a balanced set of plots and replicates and, within environment, the residual variance is expected to be constant over lines.

To provide “proof of concept”, we used a ridge-regression BLUP model with 600 randomly chosen markers. All markers were not employed in order to facilitate calculations, since matrix inversion was used for every bootstrap sample and cross-validation. With a large number of markers or individuals or for routine application, GBLUP can be computed in using iterative algorithms, but this is a numerical, as opposed to conceptual, issue. Further, 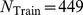 and 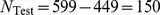; the model was trained using a grid with 10 values of 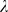: 5, 10, 50, 100, 150, 200, 250, 300, 350 and 400. All lines were present in each partition of the data into the two disjoint training and testing sets, with the process repeated at random 100 times, to estimate the cross-validation distribution. Each implementation of BGBLUP used 25 bootstrap samples and performance was evaluated via predictive correlation, predictive mean squared error and mean absolute deviation between predicted and realized phenotypes. We found (results not shown) that the “optimum” 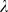 was around 50–100 in environments 1 and 2 with stronger regularization needed in environments 3 and 4. BGBLUP was slightly better than GBLUP in environment 1 near optimum regularization, and clearly better at the lower values of 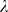 because GBLUP nearly overfitted; a similar picture emerged in environment 2. Overall, BGBLUP was better than GBLUP when 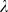 was below the optimum, sometimes slightly better at the optimum, with no difference if regularization was excessive, due to the fact that the two models were rendered effectively simpler as 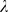 grew.

**Figure 9:**
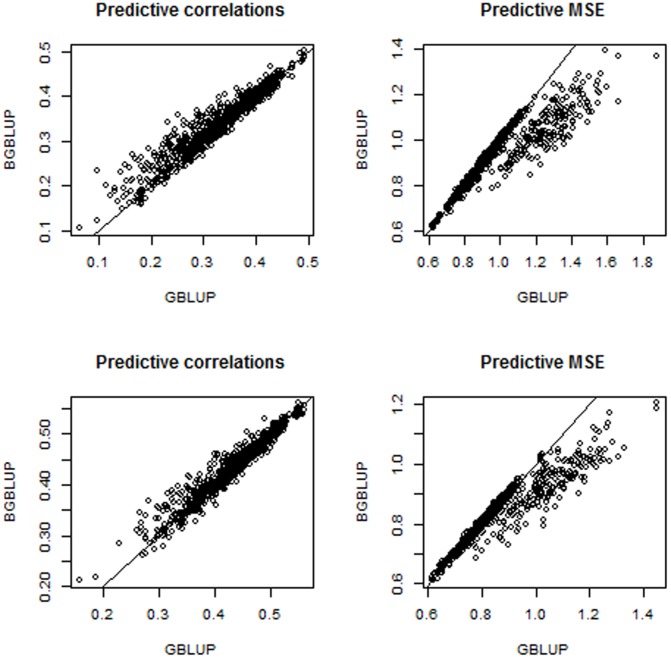
Wheat data in environments 3 and 4: predictive correlations and mean-squared errors in 1000 cross-validations (100 random partitions intro training-testing sets and 10 levels of the regularization parameter).

**Figure 10:**
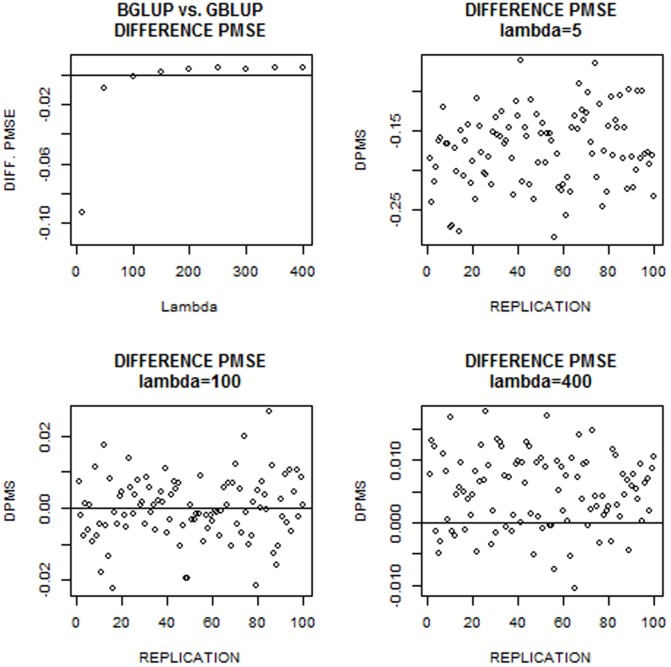
Wheat data in environment 4. Upper left panel: average differences (over 100 cross-validations) between bagged GBLUP and GBLUP for predictive correlations (PCOR), mean-squared error (PMSE) and absolute value of differences (PABS) between prediction and realization at 10 values of the regularization parameter. Upper right, lower left and lower right panels give the three metrics for lambda values of 5, 100 and 400, respectively.

The upper and lower panels of Figure 9 give results for environments 3 and 4, respectively; results for environments 1 and 2 were similar. As found with the simulated data, over the 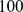 repetitions 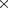
10 values of 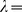
1000 comparisons, BGBLUP tended to produce larger predictive correlations and smaller mean squared errors than GBLUP. However, this superiority was not uniform and depended on whether or not the model was “under” or “over” regularized, with BGBLUP having slightly better performance in the former situation but slightly worse in the latter. This is illustrated for environment 4 in the upper left panel of Figure 10, where average differences between BGBLUP and GBLUP over the grid of lambda values are shown for predictive correlations, predictive mean square error and average absolute difference between predicted and realized values. The other panels of Figure 10 indicate that, over the 100 cross-validations, BGBLUP was better at low values of 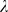, slightly better at near optimum values of 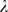 and mildly worse when regularization was extreme 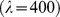. Hence, it would seem that BGBLUP performs at least as well as GBLUP unless a gross error is made in assessing the value of the regularization parameter in model training. Such a large error is unlikely if the variances are estimated from training data (unless the sample is small) or evaluated over a grid of suitable candidate values. One should be cautious about elicitations of the regularization parameter based on simple theoretical arguments that may not hold.

## Discussion

We examined whether or not bootstrap sampling in the context of GBLUP can enhance predictive ability in cross-validation. Simulation (with known or unknown QTL) and a wheat data set with grain yield information were used for this purpose. In the simulations, it was found that bagging BLUP estimates of marker effects or of genomic signal increased the slope of the regression of true marker or marked breeding value on predictor relative to what is expected under BLUP theory. When an individual was evaluated as “extreme” by GBLUP, bagging made the estimate less extreme. If the linear model entertained holds, the regression of true signal on GBLUP is expected to be 1, but the regression on BGBLUP is steeper because the latter has smaller variance. This is easy to see: if 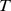 is a predictand and 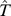 is its BLUP, then 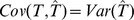 so the slope of the regression of 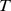 on 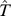 is one. Now, if 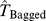 is the average of 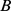 bootstrap copies of 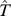, the variance of 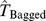 is about 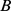 times (assuming samples are mildly correlated) smaller than that that of 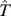, but 
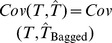
. Hence, the regression must be larger than 1.

It was also found that bagging conferred robustness to GBLUP because it is less prone to over-fitting and often delivered better predictions in terms of correlation and mean squared error even when regularization was “optimal”. At least in simulated data, BGBLUP was not inferior to GBLUP when shrinkage was beyond what it should be.

Bagging allows estimating a cross-validation prediction error mean squared error for each subject tested. In theory, given training data, GBLUP in a testing set can be computed as
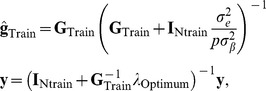
with 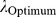 being some “optimum” value of the regularization parameter. If the problem is that of predicting a future set of records 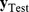 of the same individuals, the variance-covariance matrix of prediction errors (under normality assumptions) is
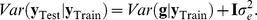
Given that the assumptions hold, there are two sources of uncertainty here. The first is uncertainty about signal (breeding value) given training data, and the second is noise variability associated with the yet to be realized observations. The model derived (expected) reliabilities of the predicted genomic value values from the training data are given by the diagonals of matrix
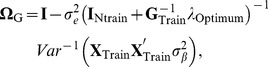
(17)where 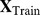 is the marker matrix in the training set. Note that (17) does not make reference at all to realized outcomes (testing set phenotypes) or to realized predictions, so using the term “accuracy” in lieu of “reliability” is misleading. While the diagonal elements of 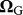 may be close to 1, this does not give assurance that predictions will be any good. The reliability matrix uses only information on marker genotypes (via the 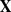 matrix) and variance components, but do not exploit information on phenotypes. Importantly, the two measures ignore model uncertainty, thus exaggerating prediction reliability relative to what would be observed in a cross-validation distribution. Model goodness of fit statistics in training data lead to expectations that seldom translate into what is observed in cross-validation (e.g., [44]) and examples of this are in a study of human height with molecular markers by [7] and [45]. The problem of developing credible individual-specific measures of reliability in cross-validation has not been solved yet [30] but a practical solution can be arrived at by use of bagging.

Let the fixed, observed, outcome (e.g., the mean of an inbred line of wheat, a daughter yield deviation of an artificial insemination bull or the phenotype of a subject) in a testing set be 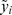, 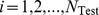, and let the prediction from GBLUP be 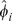, so the realized prediction error is 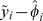. In BLUP theory, predictor and predictand (the latter with eventual realized value 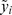) vary at random over conceptual repeated sampling, given some linear model, but here 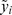 is an observed realization from an unknown process. Using bagging, 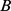 bootstrap samples of the distribution of 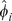 are available, so one can form the set of prediction errors 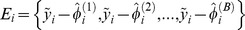 for 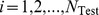. For each 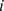, the bootstrap average squared prediction error associated with GBLUP (given 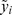, 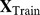 and 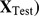 is assessed as
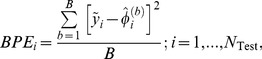
(18)noting that this squared cross-validation prediction error reflects both squared bias (unknown) and variance. Similarly, a cross-validation reliability measure can be constructed as
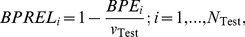
(19)where
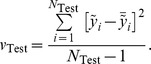


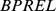
 takes values between 0 and 1 provided that 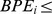 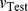, which cannot be assured unless one replaces 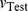 by, say, 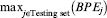. A disadvantage of 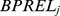 is that it does not take into account the fact that, given 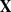, all observations are expected to have a different phenotypic variance, depending on how a genomic relationship matrix is constructed in GBLUP. Recall that GBLUP poses 
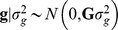
, leading to the testing set expected variance-covariance structure
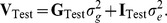
In the absence of some scaling (with the latter having consequences on the definition of 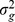) the diagonal elements of 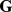 vary over individuals, so the diagonals of 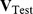 vary as well; this does not occur in a pedigree-based model if all individuals have the same level of inbreeding. One way of taking this into account is to modify the “reliability” measure (19) into
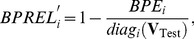
(20)where 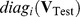 is the 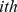 diagonal element of 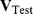.

**Figure 11:**
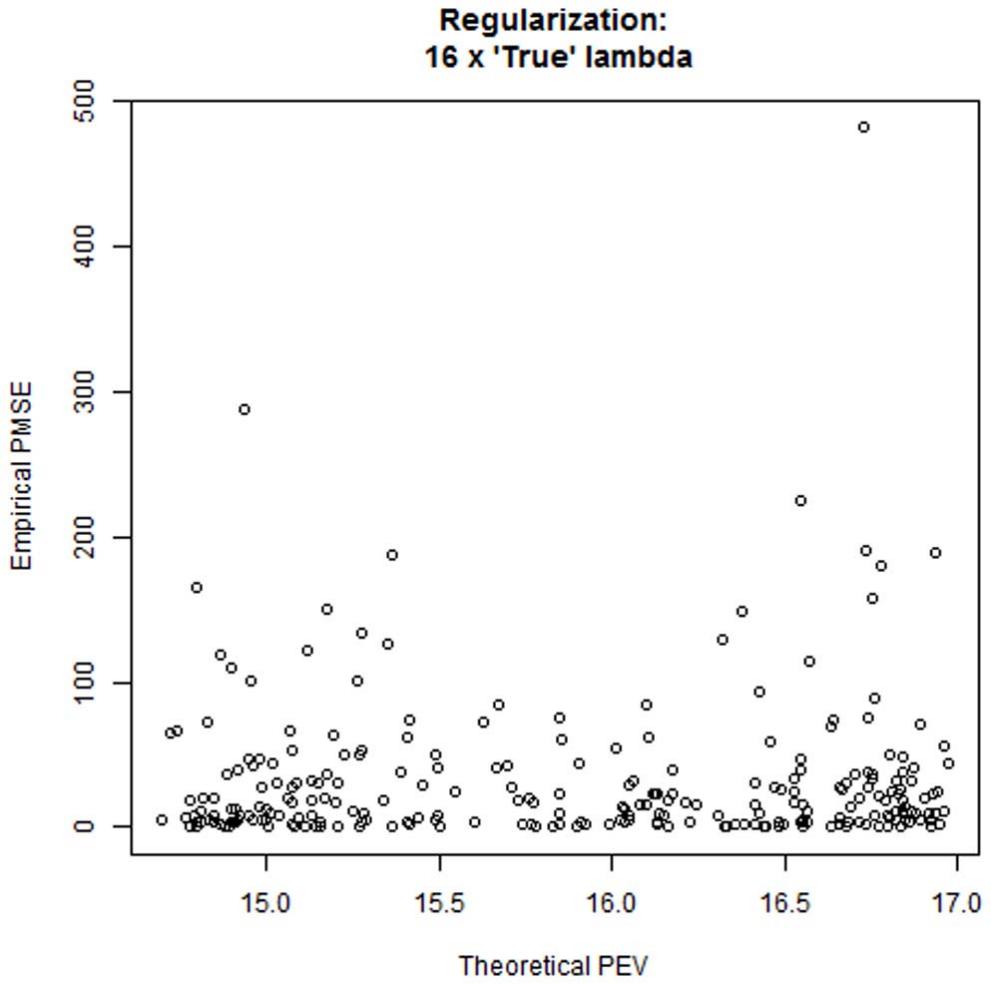
Disconnect between expected prediction error variances (theoretical PEV) and empirical bootstrap average squared prediction errors (empirical PEV) for a simulation under the settings of case study 4. True lambda = 20.

**Figure 12:**
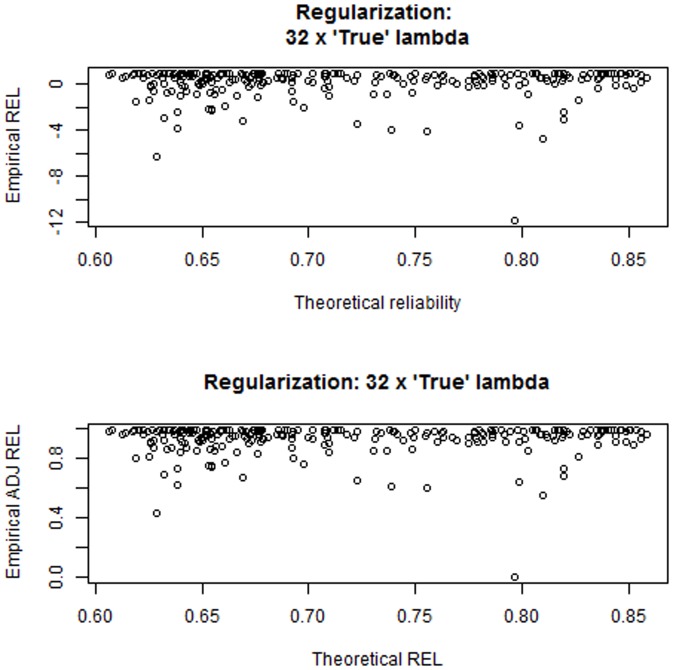
Relationship between expected reliabilities and empirical reliabilities (see text) in the top panel, and empirical adjusted reliabilities (see text) in the bottom panel.

We examined this proposal under the setting of case 4, with 100 unknown QTL, 500 binary markers, 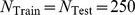. In the simulation (results not shown), we found in cross-validation that the “optimum” 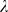 in terms of predictive correlation and mean-squared error sense was 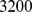. We trained the model using 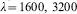 and 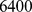 and 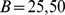 and 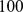. Differences in 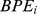 obtained with the three values of 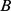 were very small and the three levels of regularization produced the same qualitative picture, with prediction mean-squared error increasing with stronger shrinkage. Figure 11 illustrates the disconnect between prediction error variances derived in the training process and bootstrap average squared prediction errors, which make use of both training phenotypes, via 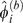 and realized values 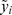. Likewise, as shown in Figure 12 the empirical 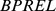 (top panel) and the adjusted reliabilities (bottom panel) are unrelated to model derived reliabilities. The adjusted reliabilities were calculated as
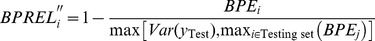
(21)with 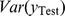 constant over the training set. When using 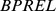 median reliability (using 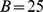) was estimated at 0.702, 0.725 and 0.722 at the three level of regularization but a few ones were negative. After the adjustment in (21) all reliabilities varied mostly between about 0.40 and slightly less than 1 and these values were unrelated to theoretical reliabilities. Predictions were quite accurate, in general (recall that the same stochastic process was used to create training and training sets).

There does not seem to be a theoretical reason leading to expect an agreement between model based reliabilities and measures of cross-validation performance, because the latter gauge different things. The theoretical reliabilities, based on a model deduced quantity, are just indicators of the amount of information in the training data set without making reference to the “goodness” of any prediction. On the other hand, 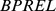 or variants thereof take into account “closeness” between prediction and realized value, with bagging enhancing the stability of the prediction. Hence, we argue that bagging is sensible because it reduces the variance of GBLUP, seemingly without hampering predictive ability, and provides a means for ascertaining the (conditional) prediction bias in a strict sense. If 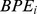 is close to 0 the squared prediction error is small, so that the prediction has a small variance, a small bias, or both. Irrespective of the cause, the cross-validation measure of reliability would be close to 1.

To discuss influences that theoretical reliability may have on predicted values in a testing set 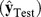, note that, when using ridge regression BLUP,
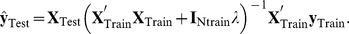
The influence training data have on predictions via GBLUP can be measured by the derivative or “hat matrix”
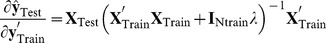
Now, the matrix of “reliabilities of marker effects” is
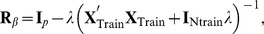
so that
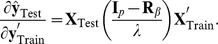
Hence, the predicted values can be seen to be less sensitive with respect to variation in training data when reliabilities (in this case of marker effects, but the same logic carries for GBLUP) increase and when 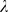 gets larger; when 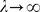 the model becomes essentially null as the effective number of parameters goes to 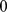. Informally, 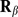 has an upper bound at 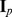 so, when reliabilities are perfect, predictions are insensitive with respect to variation in training data. However, even in this perfect case and assuming the model is correct, there is no clear connection between reliability and predictive outcome.

Bagging did reduce the variability of GBLUP predictions and, as observed in our case studies, it enhanced predictive performance when the model was “under-regularized”. When, regularization was near optimum, bagging did not improve predictive performance, but it provided a means for assessing predictive mean squared error for any individual or candidate item in a testing set. This is because bagging can emulate variation in training data sets of a given size, allowing calculation of conditional (given 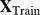, 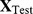 and 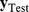) mean squared errors and of a measure of “reliability” connecting directly to predictive outcomes. These measures reflect variation in the predictor (rendered small by bagging), prediction bias and, of course, noise inherent to the fact that prediction can never be perfect. We did not find that bagging deteriorated predictive performance in any of the settings simulated, with only a slight hint in the wheat data set when regularization was excessive. As anticipated by [31] bagging helped when the predictor was more variable, due to small shrinkage. Coupled with the finding that predictive performance was not degraded otherwise, it seems that bagging confers robustness to the GBLUP prediction machine.

## Conclusions

In short, bagging ameliorated the predictive performance of GBLUP, providing a means for developing candidate-specific measures of cross-validation reliability. It is computationally intensive when one searches for an optimum value of 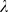 because of the simultaneous bootstrapping. In our study it seemed that 25–50 bootstrap samples were enough to attain reasonable predictions as well as stable measures of individual predictive mean squared errors. In practice, 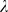 can be assessed by estimating the variance components in some data set and this may need to be done only once; the optimum 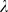 in cross-validation is often close to what one obtains from estimating 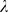 in the training set (de los Campos, personal communication), but regularization depends on the 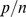 ratio, so studies from other studies with different sample sizes (even from the same population) may not provide a good guide to attain optimum regularization in a given problem.

Bagging may not be feasible for immense data sets, but a question is whether or not such huge data sets are really needed for attaining an optimal predictive performance. Typically, predictive ability increases with training set size [27], [30] but it plateaus at some point. A smaller training data set with less “molecular redundancy” than a huge data set that spans less genomic variation may deliver a better predictive performance. Perhaps the design of training data sets needs to be studied in more depth. An unresolved problem is that of reducing empirical prediction bias, which is a research area of potential interest.

## Supporting Information

Appendix S1 **Bagging may decrease predictive mean-squared error.**
(PDF)
