# Supplementary material for: Enhancing Genome-Enabled Prediction by Bagging Genomic BLUP
Source: PLoS One. 2014 Apr 10;9(4):e91693. doi: 10.1371/journal.pone.0091693 (PMC3982963; doi:10.1371/journal.pone.0091693)
Supplement: Appendix S1 — Bagging may decrease predictive mean-squared error. (PDF) [file pone.0091693.s001.pdf]

Appendix S1: Bagging may decrease predictive mean-squared error

Let the training set be $\{(y_i, \mathbf{G}'_i), i = 1, 2, ..., N_{\text{Train}}\}$, where each case is drawn from some joint distribution $\mathcal{F}$, and let $\widehat{\phi}(\mathbf{G}'_0) = \mathbf{G}'_0\ \widehat{\mathbf{g}}^*$ be the predictor at some fixed focal point $\mathbf{G}'_0$. Define the "average" predictor to be an average over the distribution of an infinite number of training sets $\mathcal{S}$

$$\widehat{\phi}_{\text{Ave}}(\mathbf{G}'_0) = E_{\mathcal{S}}\left[\widehat{\phi}(\mathbf{G}'_0)\right],$$

with the expectation taken over conceptual repetitions of training sets of size N_{Train}. For some fixed focal point and fixed output y the mean squared error is

$$E_S\left[y - \widehat{\phi}(\mathbf{G}'_0)\,|y, \mathbf{G}'_0\right]^2 = y^2 - 2yE_{\mathcal{S}}\left[\widehat{\phi}(\mathbf{G}'_0)\right] + E_S\left[\widehat{\phi}^2(\mathbf{G}'_0)\right]. \qquad (1)$$

Since $Var_{\mathcal{S}}\left(\widehat{\phi}(\mathbf{G}'_0)\right) = E_{\mathcal{S}}\left[\widehat{\phi}^2(\mathbf{G}'_0)\right] - E_{\mathcal{S}}\left[\widehat{\phi}(\mathbf{G}'_0)\right]^2 \geq 0$, it must be that $E_{\mathcal{S}}\left[\widehat{\phi}^2(\mathbf{G}'_0)\right] \geq E_{\mathcal{S}}\left[\widehat{\phi}(\mathbf{G}'_0)\right]^2$, and using this in (1) produces

$$E_{\mathcal{S}}\left[y - \widehat{\phi}(\mathbf{G}'_0)\,|y, \mathbf{G}'_0\right]^2 \geq \left(y - E_{\mathcal{S}}\left[\widehat{\phi}(\mathbf{G}'_0)\right]\right)^2 = \left(y - \widehat{\phi}_{\text{Ave}}(\mathbf{G}'_0)\right)^2. \qquad (2)$$

Taking expectations (deconditioning) over the joint distribution $\mathcal{F}$

$$E_{\mathcal{F}}E_{\mathcal{S}}\left[y - \widehat{\phi}(\mathbf{G}'_0)\,|y, \mathbf{G}'_0\right]^2 \geq E_{\mathcal{F}}\left(y - \widehat{\phi}_{\text{Ave}}(\mathbf{G}'_0)\right)^2,$$

showing that the mean-squared error of $\widehat{\phi}_{\text{Ave}}(\mathbf{G}'_0)$ over all possible input focal points-output combinations is not larger than that of $\widehat{\phi}(\mathbf{G}'_0)$. How much lower the mean squared error is depends on the inequality $E_{\mathcal{S}}\left[\widehat{\phi}^2(\mathbf{G}'_0)\right] \geq E_{\mathcal{S}}\left[\widehat{\phi}(\mathbf{G}'_0)\right]^2$ and the larger $Var_{\mathcal{S}}\left(\widehat{\phi}(\mathbf{G}'_0)\right)$ is, the more improvement averaging will produce. Now, $\widehat{\phi}_{\text{Ave}}(\mathbf{G}'_0) = E_{\mathcal{S}}\left[\widehat{\phi}(\mathbf{G}'_0)\right]$ depends on the true distribution from which the training set S is drawn. However, the bagged estimator is constructed from a bootstrap distribution that assigns probability $1/N_{\text{Train}}$ to each case in $\mathcal{S}$. [31] stated that if the predictor is unstable, the improvement from averaging (aggregation) may be sizable, but if the predictor is stable the bootstrap approximation to the distribution may be inaccurate. Unfortunately, "stability" is a vague and relative concept. He concludes:

> *"Bagging goes a long way towards making a silk purse out of a sow's ear, especially if the sow's ear is twitchy. It is a relatively easy way to improve an existing method..."*
